# Barriers to Quantitative Electron Probe X-Ray Microanalysis for Low Voltage Scanning Electron Microscopy

**DOI:** 10.6028/jres.107.049

**Published:** 2002-12-01

**Authors:** Dale E. Newbury

**Affiliations:** National Institute of Standards and Technology, Gaithersburg, MD 20899-8371

**Keywords:** electron probe x-ray microanalyzer, energy dispersive spectrometry, low voltage microanalysis, scanning electron microscope, wavelength dispersive spectrometry, x-ray spectrometry

## Abstract

Low voltage x-ray microanalysis, defined as being performed with an incident beam energy ≤5 keV, can achieve spatial resolution, laterally and in depth, of 100 nm or less, depending on the exact selection of beam energy and the composition of the target. The shallow depth of beam penetration, with the consequent short path length for x-ray absorption, and the low overvoltage, the ratio of beam energy to the critical ionization energy, both contribute to minimizing the matrix effects in quantitative x-ray microanalysis when the unknown is compared to pure element standards. The low beam energy restricts the energy of the atomic shells that can be excited, forcing the analyst to choose unfamiliar shells/characteristic peaks. The low photon energy shells are subject to low fluorescence yield, so that the peak-to-continuum background is reduced, severely limiting detectability. The limited resolution of semiconductor energy dispersive spectrometry results in frequent peak interference situations and further exacerbates detection limits. Future improvements to the x-ray spectrometry limitations are possible with x-ray optics-augmented wavelength dispersive spectrometry and microcalorimeter energy dispersive spectrometry.

## 1. Introduction: Advantages of Low Voltage X-Ray Microanalysis

Electron probe x-ray microanalysis, as performed in the conventional beam energy range, generally defined as 10 keV ≤ beam energy (*E*_0_) ≤ 30 keV, provides a powerful tool for spatially-resolved analysis [1]. Spatial resolution on a lateral and depth scale of approximately 1 m to 5 μm can be achieved, depending on the exact choice of beam energy and specimen composition. By choosing the beam energy in the conventional range, a satisfactory analytical x-ray peak, from the K, L, or M shells, can be found for elements with Z ≥ 4 (Be). For some elements, more than one family of characteristic peaks can be selected, e.g., for copper, both the K- and L-family peaks can be excited, while for gold, the L- and M-family peaks are accessible. “Satisfactory peak” means that at least one characteristic peak for an element can be excited with sufficient peak-to-background (characteristic relative to the x-ray continuum) so that elemental concentrations in the major (C > 0.1 mass fraction) and minor (0.01 ≤ C ≤ 0.1) ranges can be measured in reasonable counting times, of the order of 100 s. Trace levels (C < 0.01 mass fraction) as low as a mass fraction of 10^−4^ [100 ag/pg, or 100 parts per million (ppm))] can be measured providing the spectrometry technique employed has sufficient spectral resolution. For electron probe x-ray microanalysis (EPMA), this requirement has been traditionally met through the use of wavelength dispersive spectrometry (WDS). Energy dispersive x-ray spectrometry, with its poorer spectral resolution, is usually restricted to concentration levels above 0.01 mass fraction for quantitative measurements with a limit of detection of 0.001 mass fraction, especially if peak interference occurs.

For a particular specimen composition, the spatial resolution of electron-excited x-ray microanalysis improves as the incident beam energy is reduced. Interest in quantitative x-ray microanalysis at low beam energy (*E*_0_ ≤ 5 keV) has been greatly stimulated by the development of the high resolution field emission gun scanning electron microscope (FEG-SEM). The principal rationale for this interest is the possibility of achieving x-ray spatial resolution similar in scale to high performance FEG-SEM electron imaging. Analytical resolution depends upon the x-ray range, which follows a relationship of the form [2]:

$$R(\mathrm{nm})=\left[27.6A/\left(\rho Z^{0.89}\right)\left({E_{0}}^{1.67}-{E_{c}}^{1.67}\right)\right]$$ (1)

where *E*_0_ is the incident beam energy (keV), *E*_c_ is the critical excitation energy for the characteristic x rays of interest (keV), *A* is the atomic weight (g/mol), *Z* is the atomic number, and *ρ* is the density (g/cm^3^). The spatial resolution advantage of low voltage operation is seen in Tables 1 and 2. Based on Eq. (1), Table 1 lists the range of x-ray production for Cu K (*E*_c_ = 8.98 keV) in various matrices (i.e., Cu as a trace constituent in various matrices) in the conventional beam energy range (10 to 30 keV). For operation in the conventional range with the requirement that the overvoltage (*U* = *E*_0_/*E*_c_) must be ≥ 2; *E*_0_ > 19 keV), the Cu K-shell x-ray production range ranges from approximately 5 μm for a light element matrix like carbon. For intermediate atomic numbers, the Cu K-range is approximately 1 μm while for high atomic number elements the range decreases toward 500 nm. By operating in the low voltage regime and utilizing the Cu L shell instead of the Cu K shell, Table 2, the x-ray production range for an overvoltage of *U* ≈ 2.5 (*E*_0_ = 2.5 keV) drops to about 100 nm for carbon and to 20 nm for gold, an improvement in spatial resolution of approximately a factor of 50 in linear dimensions, or about 10^5^ in volume. This level of resolution approaches that achieved by high voltage analytical electron microscopy of thin sections (≈100 nm thickness) but with the added advantage that specimens can be examined in the FEG-SEM without thinning. Often specimens can be examined in the FEG-SEM without any special preparation at all, especially if the combination of a FEG-SEM and an environmental/variable pressure SEM is utilized.

A second positive aspect of low voltage microanalysis is the dependence upon beam energy of the matrix or inter-element effects, atomic number (scattering and stopping power), absorption, and secondary x-ray fluorescence, which must be considered in quantitative analysis. When calculated as a correction factor between multi-element unknown and pure element standards, all of the matrix correction factors tend toward unity as the beam energy is lowered into the low voltage regime. X-ray absorption, which is often a major source of experimental error at conventional beam energies, is much less significant under low voltage analysis conditions because of the greatly reduced absorption path length that results from the decrease in the beam penetration range and the exponential dependence of absorption on path length through the material.

## 2. What Are We Actually Measuring?

When operating in the conventional beam energy range, the x-ray generation and sampling depth are generally of the order of 1 μmr. While such a range is extremely shallow compared to the millimeter to centimeter thickness of bulk specimens, it is nevertheless sufficiently deep that the excited volume predominantly samples the bulk composition in most cases. Although most pristine materials, when exposed to moist atmosphere, react to form native surface oxide layers, such layers tend to have thicknesses in the range of only a few nanometers, e.g., for aluminum, about 4 nm of aluminum oxide (Al_2_O_3_) rapidly forms on a bare metal surface. When the x-ray generation range is 1000 nm to 2000 nm, a surface layer with a thickness of a few nanometers of oxide causes a negligible deviation (less than a percent) in the measured intensity from that produced by a target with an ideal bare surface.

In low voltage microanalysis, the sampling situation is much more complicated. With the much shallower ranges listed in Table 2, a surface layer of several nanometers in thickness constitutes a much larger fraction of the sample. The analytical strategy needed to deal with this situation to achieve quantitative analysis must be based upon treating every specimen as a layered target. Measurement at a progressive sequence of beam energies can provide the critical information necessary to separately characterize a layer or multiple layers on a substrate.

## 3. Limitations to Low Voltage Microanalysis: X-Ray Spectrometry Problems

### 3.1 WDS or EDS?

For well over two decades, low beam energy x-ray microanalysis has been an established capability of the electron probe microanalyzer (EPMA) equipped with wavelength dispersive x-ray spectrometry (WDS). WDS has the spectral resolution performance necessary to solve the numerous spectrometry problems that arise in the low photon energy range, as discussed below. Unfortunately, the overall efficiency of the WDS is low due to its small solid angle of acceptance, losses in the diffraction process, and most significantly, the time serial nature of its spectral collection. The EPMA is capable of delivering high beam current, 100 nA to 1000 nA, in the focused probe, which can compensate for the low WDS efficiency. This enables useful measurements, including wavelength scans, to be made within practical time periods. Ideally, the WDS could be adapted to the FEG-SEM to serve the same role in low beam energy x-ray microanalysis. However, the limiting beam current in the high brightness, cold field emission gun SEM is typically in the range 100 pA to 1 nA, a level that increases the time penalty for WDS measurements by a factor of 100 to 10 000 compared to the high current EPMA. An attractive alternative is a FEG-SEM equipped with a Schottky gun that can produce higher currents (100 nA to 500 nA), a more compatible beam current situation for the use of WDS. However, the Schottky gun has inherently lower brightness than the cold field emission gun, and therefore poorer electron-optical performance (beam current into a specific probe size) at low beam energy.

With this severe limitation imposed on utilizing conventional WDS in low beam current instruments, the usual choice for low beam energy x-ray spectrometry in high resolution SEMs necessarily becomes the semiconductor energy dispersive spectrometer with a monolithic silicon crystal (Si-EDS). Si-EDS has sufficient geometric collection efficiency and quantum efficiency to operate with the picoampere to low nanoampere beam currents of the FEG-SEM. Unfortunately, the optimum resolution performance of approximately 125 eV to 129 eV (full peak width at half the maximum peak intensity, FWHM) at MnKα (5890 eV) leads to significant limitations in x-ray spectrometry of low energy photons. Moreover, when photons below 1 keV are to be measured, the design of the EDS must be optimized to yield the best possible performance in this range. Low energy photons have a relatively short range and are therefore absorbed near the front face of the detector where they are particularly vulnerable to “incomplete charge collection” artifacts. These artifacts include peak shift and resolution degradation due to distortion on the low energy side of the peak. New detector designs and improved detector processing to address the problem of incomplete charge collection have improved the resolution performance in the low photon energy region below 1 keV. Resolution of approximately 60 eV at C K (282 eV) can now be achieved, compared to 90 eV to 100 eV obtainable with “old technology” detectors, despite equivalent resolution performance at MnKα, where the x rays are absorbed deeper into the detector crystal [3].

### 3.2 Peak Interference Problems

**Fig. 1:**
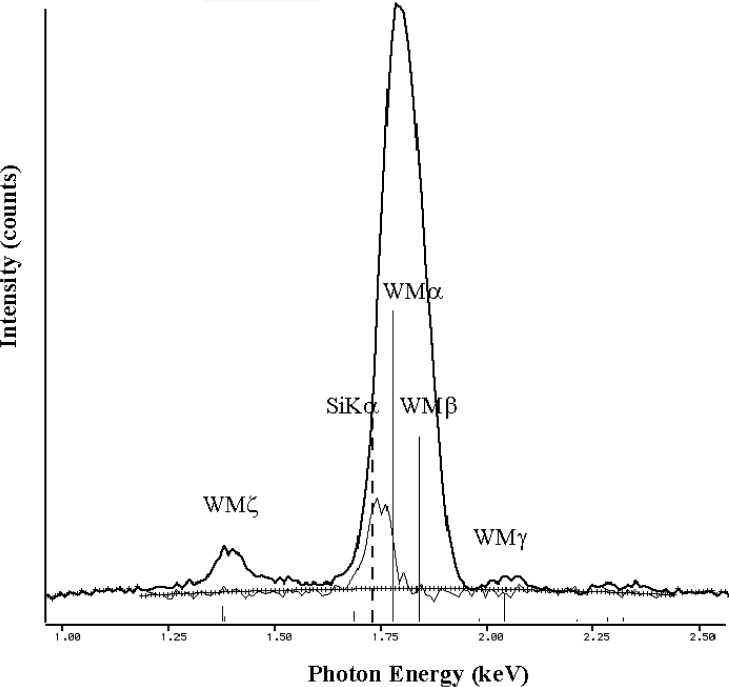
Si-EDS (FWHM = 129 eV at MnKα, 61 eV at C K) spectrum of WSi_2_ showing interferences (SiKα at 1.740 keV with WMα at 1.775 keV) revealed by W-peak fitting with multiple linear least squares. The thin-line trace shows the residuals after removal of the W-peak structure.

**Fig. 2:**
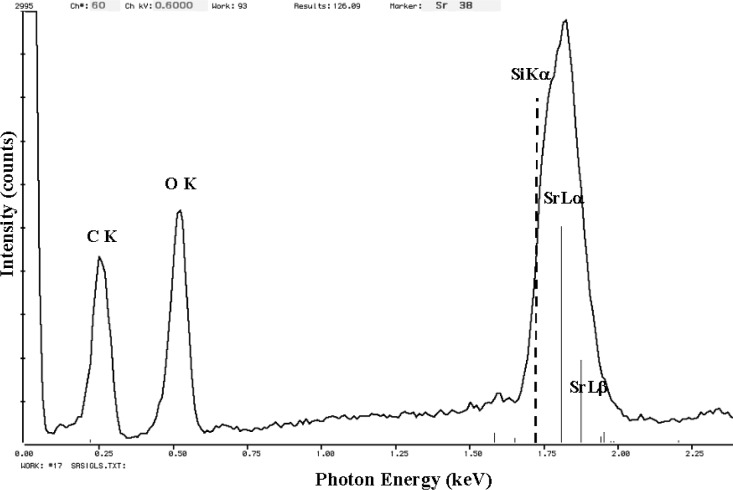
Si-EDS (FWHM = 129 eV at MnKα, 61 eV at C K) spectrum of strontium orthosilicate, SrSiO_4_ (SiKα at 1.740 keV with SrLα at 1.806 keV),

**Fig. 3:**
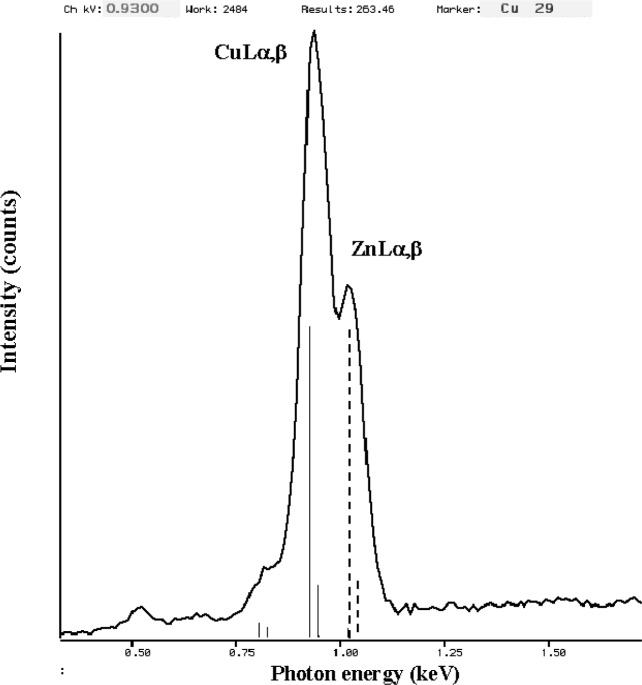
Si-EDS (FWHM = 129 eV at MnKα, 61 eV at C K) spectrum of brass CuZn (CuLα at 0.928 and ZnLα at 1.009 keV)

**Fig. 4:**
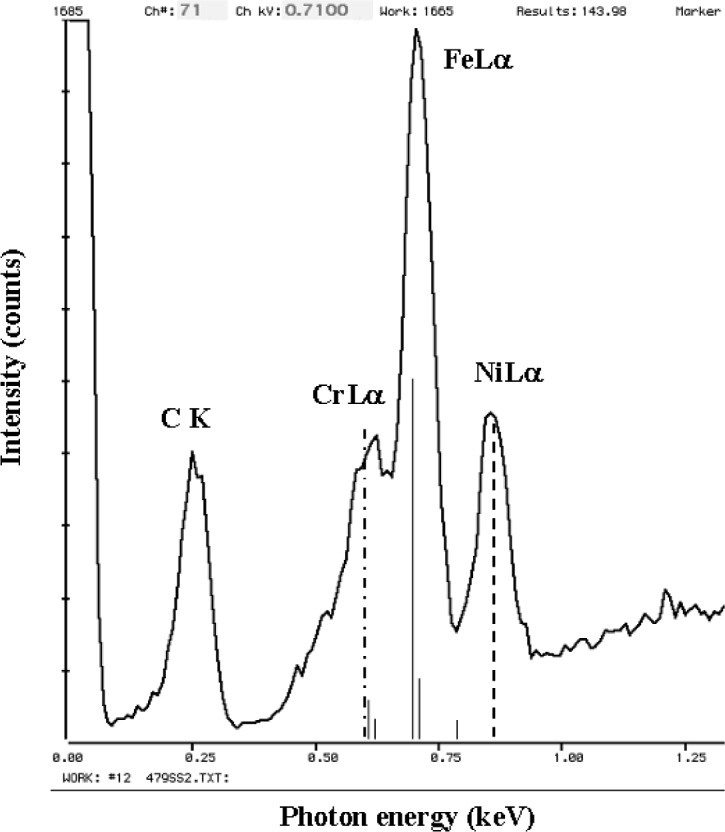
Si-EDS (FWHM = 129 eV at MnKα, 61 eV at C K) spectrum of stainless steel, CrFeNi (CrLα at 0.571 keV; FeLα at 0.711 keV; and NiLα at 0.849 keV).

The relatively broad resolution response of Si-EDS compared to the natural width of characteristic x-ray peaks leads to significant peak interference problems in the low photon energy range. Examples of technological materials which give rise to severe interferences in Si-EDS spectra in the low voltage analysis regime include tungsten silicon compounds, e.g., W_5_Si_3_, shown in Fig. 1 (SiKα at 1.740 keV with WMα at 1.775 keV); strontium orthosilicate, SrSiO_3_ in Fig. 2 (SiKα at 1.740 keV with Sr Lα at 1.806 keV); brass, 70Cu-30Zn in Fig. 3 (CuLα at 0.928 and ZnLα at 1.009 keV); and stainless steel, Fe-18Cr-10Ni in Fig. 4 (CrLα at 0.571 keV; FeLα at 0.711 keV; and NiLα at 0.849 keV). While the individual spectral components of these interferences possibly can be separated by mathematical peak fitting procedures, such as multiple linear least squares fitting, the stability of such procedures becomes questionable when the relative peak height differences become large or when limited counting statistics are available.

**Fig. 5:**
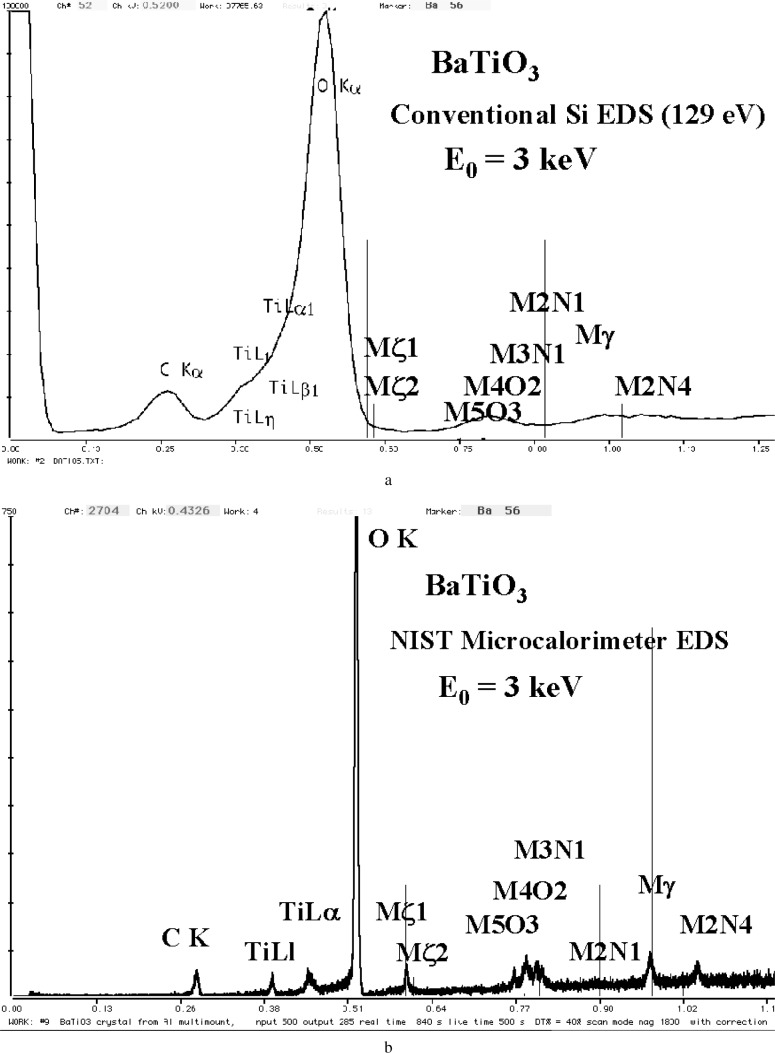
(a) Si-EDS (FWHM = 129 eV at MnKα, 61 eV at C K) spectrum of BaTiO_3_ excited in the low voltage analysis region with E_0_ = 3 keV; (b) spectrum of BaTiO_3_ as measured with the microcalorimeter EDS.

**Fig. 6:**
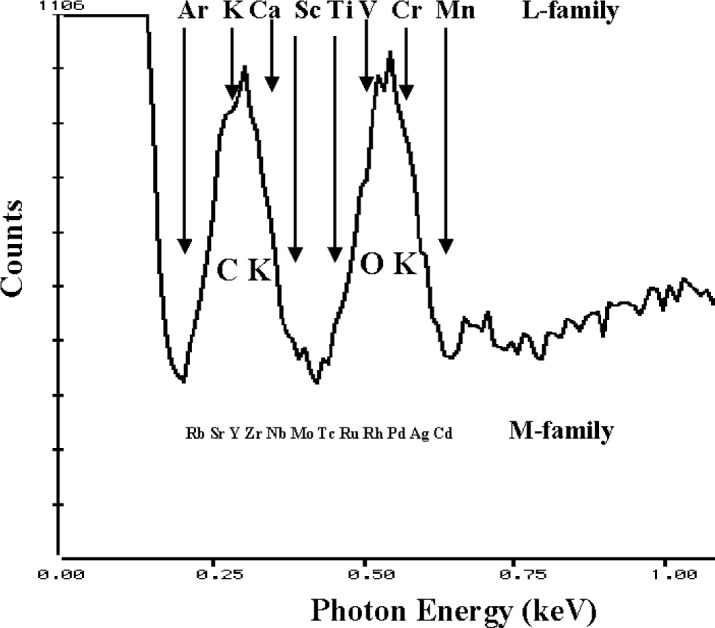
The C K and O K region of the x-ray spectrum showing interferences with L- and M-family peaks of heavy elements.

The interference situation is especially severe when we must select combinations of elements that involve the fluorescence yields of the L and M shell peaks relative to the K-shell peaks. Although the K-shell peaks of the low atomic number elements have low fluorescence yields compared to higher energy K-shell photons, the yields for the L and M-shell peaks in this energy region are much lower still. This problem can be seen in Fig. 5(a) for BaTiO_3_, where the Ba M-family peaks are barely visible against the background despite the large fraction of Ba in the compound (0.589 mass fraction). The Ti L-peaks are unresolved and only form a shoulder on the O K peak, despite the fact that Ti is of similar mass fraction (0.205) as oxygen (0.206). Since many materials of interest contain oxygen, and carbon is often present as a contaminant, it is interesting to consider interferences that occur in the C K to O K region of the spectrum. Figure 6 shows this region and the locations of the main peaks from various L- and M-family elements that would potentially suffer interference in this region. As can be seen in Fig. 5(a) and Fig. 6, a relatively small mass fraction of carbon and oxygen could cause severe interference with the L- and M-family peaks because of the large difference in relative fluorescence yields.

### 3.3 Consequences of Low Overvoltage: Limitations on Available Analytical Peaks

**Fig. 7:**
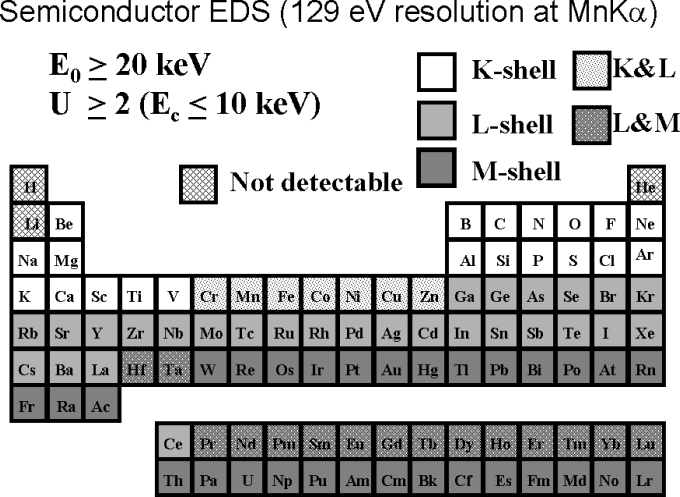
Periodic Table showing typical choices of atomic shells for operation in the conventional beam energy range, *E*_0_ = 10 keV to 30 keV.

In EPMA analysis performed in the conventional beam energy range, good operating strategy involves selecting an incident beam energy, *E*_0_, that produces an overvoltage, *U* = *E*_0_/*E*_c_ = 2, where *E*_c_ is the critical excitation edge energy, for the highest energy shell of interest [1]. Such an overvoltage generally results in an adequate value of the characteristic-to-continuum ratio, the peak-to-background (*P/B*), to permit detection of concentration levels to trace levels (C < 0.01 mass fraction) for most elements with Si-EDS and with reasonable counting times (*t* < 500 s). This strategy provides at least one useful characteristic peak for all elements with *Z* ≥ 4 (Be), and for many elements, more than one shell can be excited, e.g., K and L for Cu, or L and M for Au. Thus, the analytical strategy for shell selection for a particular beam energy e.g., *E*_0_ = 20 keV, in the conventional range can be presented in the form of an annotated Periodic Table, as shown in Fig. 7.

**Fig. 8:**
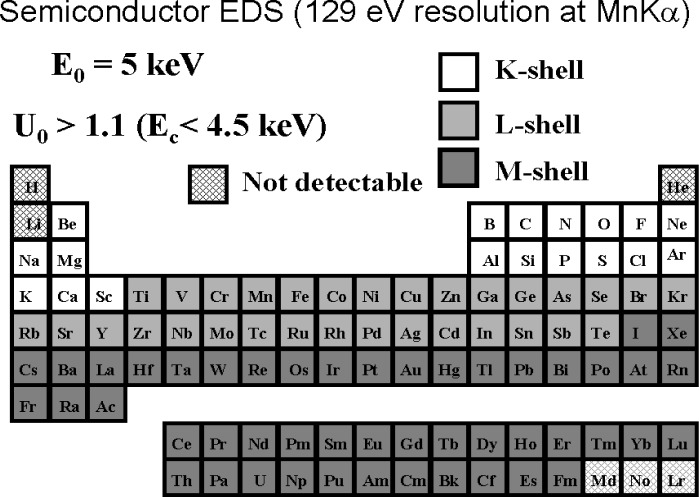
Periodic Table showing choice of atomic shells available for operation in the low beam energy range, *E*_0_ = 5 keV and *U* = 1.1.

**Fig. 9:**
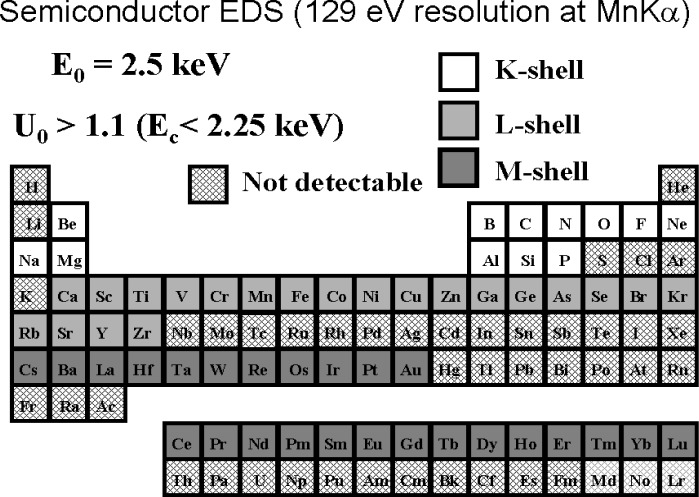
Periodic Table showing choice of atomic shells available for operation in the low beam energy range, *E*_0_ = 2.5 keV and *U* = 1.1. Note significant loss of elements that can be effectively measured.

When the low voltage microanalysis regime is considered at the upper limit beam energy of 5 keV, the overvoltage limits access to the more energetic shells of the intermediate and high atomic number elements, as shown in Fig. 8 [4]. Changes must now be made in the selection of shells for some elements compared to the selection for conventional analysis, e.g., Ti-L instead of Ti-K; Ba-M instead of Ba-L. Moreover, overvoltages as low as *U* > 1.1 must be accepted to obtain a peak for measurement for some elements, but with an inevitable decrease in the *P/B*, and consequently sensitivity, that can be obtained. As the beam energy is lowered further into the low voltage microanalysis regime, significant portions of the Periodic Table become inaccessible just on the basis of poor excitation, as shown in Fig. 9 for *E*_0_ = 2.5 keV and *U* = 1.1.

### 3.4 Restriction to Shells With Low Fluorescence Yield

The poor efficiency of characteristic x-ray excitation due to low overvoltage is not the only impediment to low voltage microanalysis. As the beam energy is reduced below the conventional operational range into the low voltage regime (*E*_0_ ≤ 5 keV), the energy of the shells that can be ionized necessarily falls, so that access to the deeper shells of the heavier elements is reduced or eliminated. The accessible L and M shells that can be efficiently ionized for the intermediate and heavy elements are often subject to low fluorescence yield (the fraction of ionizations that result in photon emission). Low peak fluorescence yield translates into a low value of the generated characteristic-to-continuum (*P/B*) ratio. After convolution with the EDS resolution broadening function, the measured *P/B* ratio is even further reduced.

*P/B* is a critical term in determining the limit of detection. The intensity of characteristic x-rays depends on the overvoltage, *U*, which is the ratio of the incident beam energy, *E*_0_, to the excitation edge energy, *E*_c_, for the characteristic peak of interest: Experimentally, the measured characteristic intensity (normalized for beam current and detector solid angle angle) is found to follow a relation of the form:

$$P\sim {\left(U-1\right)}^{n}$$ (2a)

$$U=E_{0}/E_{c}$$ (2b)

where the exponent n is typically found in the range 1.3 to 1.7 [1,5]. The background of the electron-excited spectrum consists of (ideally) the x-ray bremsstrahlung, which forms a continuous distribution from the lowest photon energy up to the energy of the incident beam, *E*_0_, as it enters the target. The x-ray continuum intensity depends upon the average atomic number, *Z*, and the continuum photon energy, *E_v_*, of interest:

$$B\sim Z\left(E_{0}-E_{v}\right)/E_{v}$$ (3a)

When we consider the background directly under a characteristic peak, then *E_v_* = *E*_P_. Although *E*_P_ < *E*_c_ for a given element, we can approximate *U* = *E*_0_/*E*_c_*~ E*_0_/*E_v_*. With this approximation, Eq. (3a) can be rewritten as:

$$B\sim Z\left(E_{0}-E_{v}\right)/E_{v}\sim Z\left(U-1\right)$$ (3b)

The peak-to-background ratio, *P/B*, can then be described as:

$$P/B={\left(U-1\right)}^{n}/Z\left(U-1\right)=\left(1/Z\right){\left(U-1\right)}^{n-1}$$ (4)

**Fig. 10:**
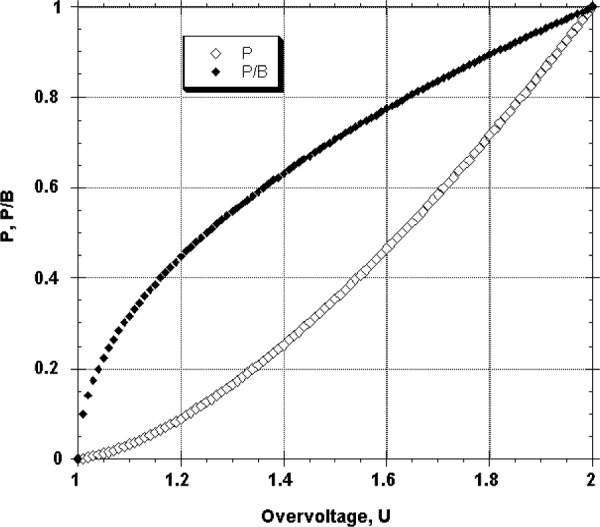
Behavior of the peak (*P*) and peak-background (*P/B*) ratio as a function of overvoltage

The behaviors of the peak and peak-to-background in the low overvoltage range predicted by Eqs. (2) and (4) are shown in Fig. 10. The plot of *P/B* vs *U* shows a sharp decrease in *P/B*, which is an important factor in the limit of detection, as the overvoltage decreases toward unity.

The consequence of a low fluorescence yield is illustrated in Fig. 5(a) for the peaks that constitute the Ba M-family, measured with a Si-EDS with resolution performance of 129 eV at MnKa and 60 eV at C K. The L-shell is the usual choice for an analytical peak for barium in the conventional beam energy range, but the L-shell is not accessible in the low beam energy range, since the BaL_3_ *E*_c_ is 5.247 keV. The M-shell peaks illustrated in Fig. 5(a) show a low *P/B* despite the presence of Ba in the compound at the level of 0.588 mass fraction.

Low *P/B* has a deleterious impact on the limit of detection. The concentration limit of detection can be estimated from the spectrum of Fig. 5(a) using the Ziebold (1967) expression (originally derived for a pure element but modified to accommodate measurements on a compound) [6]:

$$C_{\mathrm{DL}}>\left(3.29\;a\right)/{\left[n\;\tau (P/Ci)(P/B)\right]}^{1/2}$$ (5)

where *a* is the constant in the Ziebold-Ogilvie hyperbolic equation [7] linking the *k*-value (where *k* = peak intensity of unknown/peak intensity of standard) and the concentration *C*, *n* is the number of measurements, *τ* is the measurement time, *P* is the peak counting rate, *C*_i_ is the concentration (mass fraction) of the element of interest in the compound, and (*P/B*) is the peak-to-background on the standard. Based upon the measurement parameters for the spectrum in Fig. 5(a) (the peak counting rate was taken at a deadtime of 20 %), and estimating *a* = 1, the values in Table 3 are predicted for concentration limit of detection, *C*_DL_ (mass fraction), of an element similarly excited as barium. *C*_DL_ is calculated as a function of measurement time (for a single measurement) for the most prominent M-peak observed for Ba (the M_3_N_1_ transition).

Thus, under low voltage analysis conditions with conventional Si-EDS, the detection situation for an element excited with the efficiency of the barium M-shell can only be measured across the full major constituent range (*C* > 0.1 mass fraction) with a counting interval of 1000 s and 20 % deadtime. To detect a constituent at the middle of the minor range, an accumulation time of 10 000 s or more would be necessary. Trace level measurements (*C* < 0.01) would be impractical with such low excitation.

It should also be noted that the relative peak abundances indicated by the lines (derived from the relative peak intensities reported in the Bearden Tables) strongly disagree with the relative heights of the peaks observed experimentally. For example, the strongest peak appears to be associated with the M_3_N_1_ transition, rather than the M_γ_ peak (the transition M_3_N_4,5_) reported in the references.

## 4. Possible Solutions to X-Ray Spectrometry Problems

Two emerging technologies may significantly improve the measurement situation for low energy photons in the 100 eV to 5 keV range: (1) WDS augmented with x-ray optics and (2) microcalorimetry.

### 4.1 WDS/X-Ray Optics

The WDS is constrained in two important ways relative to the low intensity x-ray source available in FEG-SEM instruments operating in the low voltage regime: (1) low efficiency due to the small solid angle of collection and the inefficiency of the diffraction process for some diffractors ; and (2) the narrow energy bandpass of a few eV, meaning that most of the excited spectrum is lost at the diffractor and never reaches the detector. Instrumentation advances have been proposed and/or made to at least partially overcome these limitations.

1. Increasing the WDS efficiency: The efficiency of the WDS has been improved through incorporation of capillary x-ray optics [8] (Agnello et al., 1997). In their design, which is now available commercially, a capillary optic bundle tapered on one end is used to collect x rays over a large solid angle, conduct the x rays along the inside surface of the capillaries by total external reflection, and then present a parallel beam of x rays to a flat crystal diffractor. The flat diffractor scatters with a high degree of efficiency into a gas proportional counter. This system has realized a gain of a factor as high as 50 compared to a conventional curved crystal WDS for characteristic x rays in the energy range from 100 eV to 1.8 keV.
2. Parallel detection in WDS: The greatest loss in available information occurs because of the time serial nature of the WDS measurement. Moreover, peak top measurements alone are insufficient. For accurate intensity measurement, the background under the peak must be estimated, usually by directly measuring the background on either side of the peak. Fiori et al. observed that a dispersed image of a WDS peak could be obtained by taking advantage of the focusing action of a curved crystal wavelength dispersive spectrometer [9]. By placing both the electron-excited source and a suitable multichannel detector inside the Rowland circle while maintaining the diffractor in its conventional location on the circle, a dispersed image of the x-ray peak and the nearby background could be obtained. Such a parallel measurement of the peak would improve efficiency by permitting detection of all characteristic photons that comprise the peak, which would increase sensitivity, and additionally, it would reduce the significance of chemical effects that alter peak shape, since the full peak shape could be recorded for every measurement. Moreover, for quantitative analysis, the critical step of background removal could be made more accurate by fitting a large number of channels of data compared to conventional WDS practice of on-peak, off-peak measurements.

### 4.2 Energy Dispersive X-Ray Spectrometry by Microcalorimetry

The development of the microcalorimeter energy dispersive x-ray spectrometer (μcal-EDS) is one of the most promising approaches for solving many of the spectrometry problems that limit low voltage microanalysis [10]. The μcal-EDS operates on the principle of absorbing an x-ray photon in a metal target and measuring the resulting temperature rise. The μcal-EDS operates at extremely low temperature, 100 mK, and the detector makes extensive use of superconducting cryoelectronics for the critical thermometry that constitutes the measurement of photon energy. The principal characteristics of the microcalorimeter-EDS as related to low voltage microanalysis can be summarized as follows [11].

**Fig. 11:**
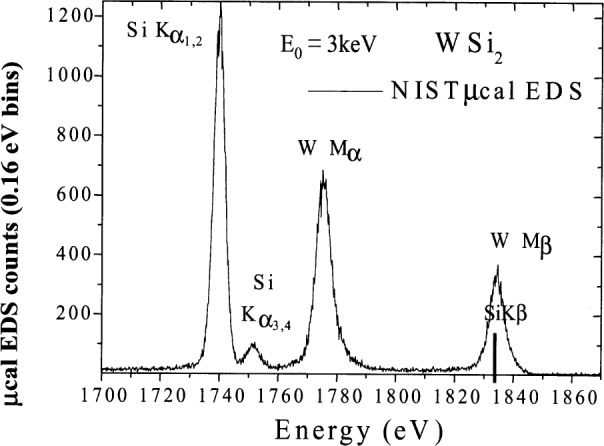
Comparison of resolution vs photon energy for various spectrometers.

Resolution: The microcalorimeter-EDS provides resolution performance, 2 eV to 10 eV, similar to that of WDS but with operation in an energy dispersive mode. Figure 11 shows a plot of resolution for a variety of spectrometers: Si-EDS, WDS, and the microcalorimeter-EDS. The resolution of the microcalorimeter-EDS is similar to the WDS over most of the photon energy range. For the very low energy photons below 500 eV for which layered synthetic materials (LSM) are the leading choice as WDS diffractors, the microcalorimeter-EDS actually has a significant resolution advantage, 2 eV to 3 eV compared to 10 eV to 14 eV for LSMs.

**Fig. 12:**
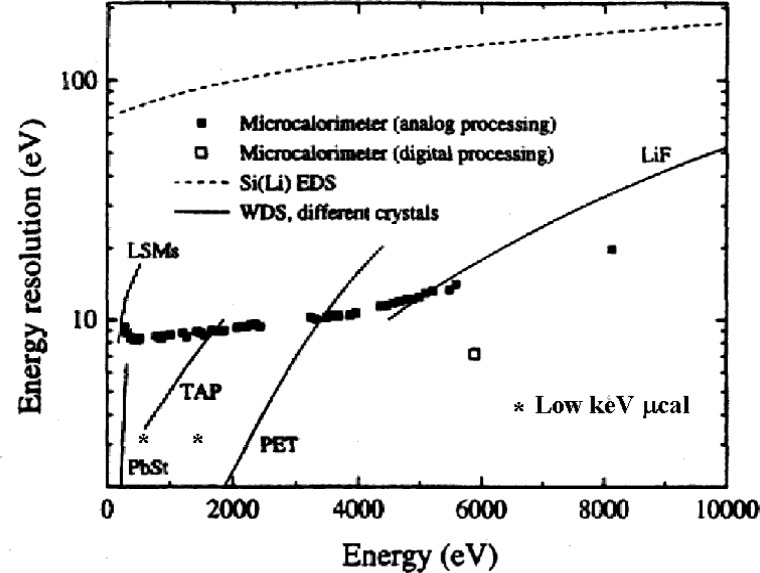
Microcalorimeter energy dispersive x-ray spectrum of WSi_2_, showing separation of the SiK peak from the W-M family x-ray peaks.

**Fig. 13:**
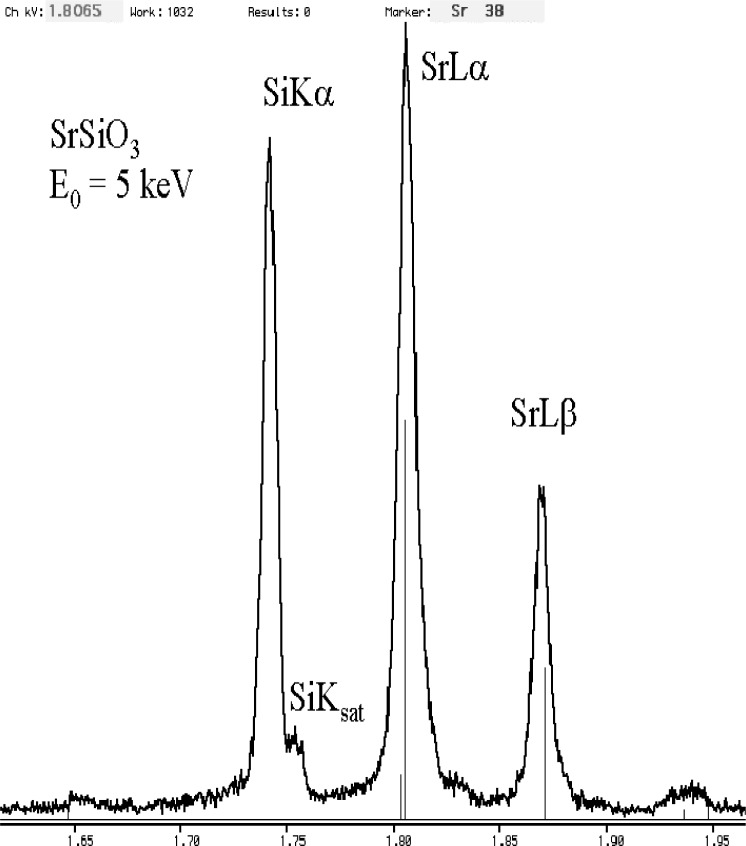
Microcalorimeter energy dispersive x-ray spectrum of SrSiO_3_, showing separation of the SiK peak from the Sr-L family x-ray peaks.

**Fig. 14:**
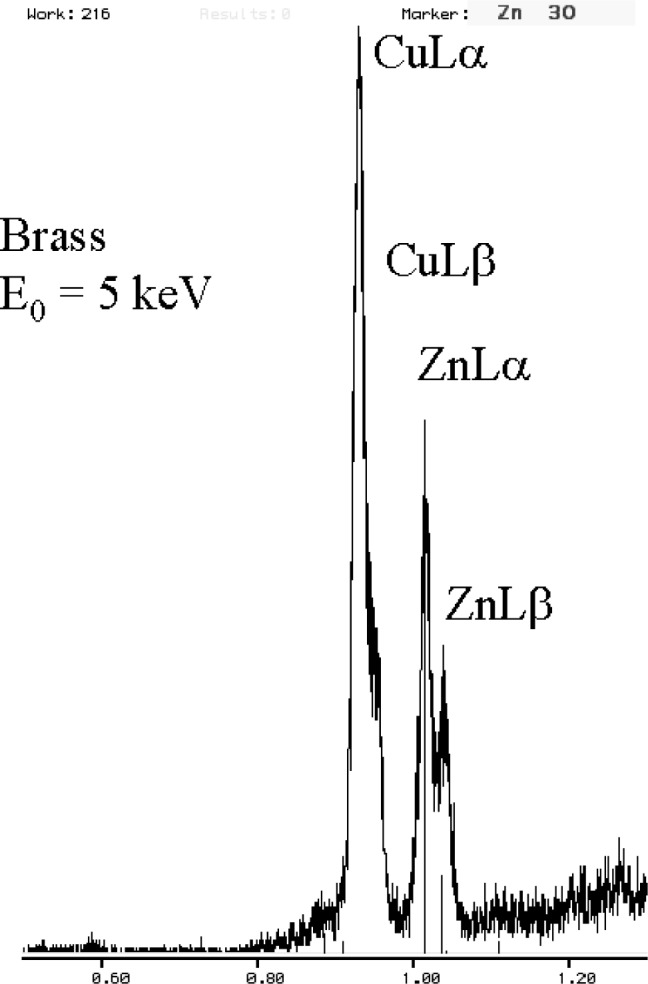
Microcalorimeter energy dispersive x-ray spectrum of brass, showing separation of the CuL family peaks from the ZnL family peaks.

**Fig. 15:**
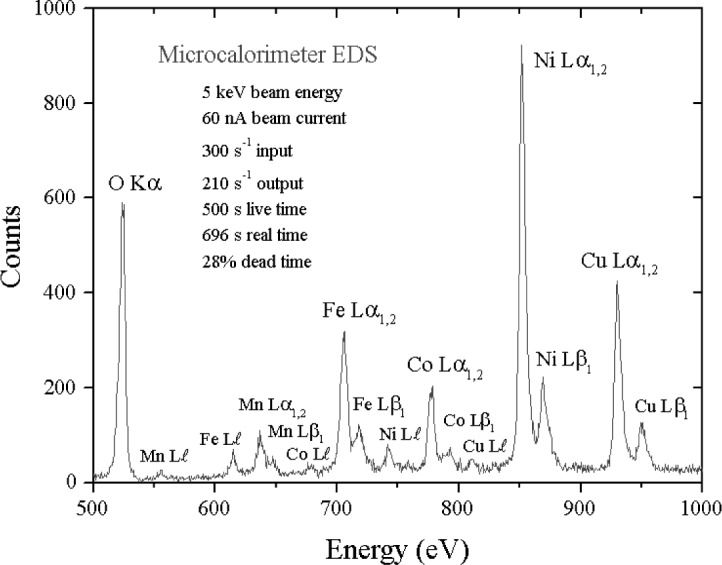
Microcalorimeter energy dispersive x-ray spectrum of a complex alloy, showing separation of the L-family peaks for Mn, Fe, Co, Ni and Cu.

This resolution performance of the microcalorimeter-EDS is best appreciated by inspecting examples of spectra for materials of interest. Consider the difficult examples for conventional Si-EDS presented in Figs. 1–4. Figures 12–15 show the performance of the microcalorimeter-EDS on the same or very similar materials: WSi_2_, SrSiO_3_, brass, and a mixture of the transition metals. In all cases, the major and most minor peaks of the elements are well resolved, permitting unambiguous elemental identification for qualitative analysis, providing well separated peaks for accurate peak intensity measurement necessary for quantitative analysis, and yielding high *P/B* ratios to achieve low levels of detection. The improvement in the spectrometry situation is well illustrated by the case of BaTiO_3_ shown in Fig. 5(b) where the microcalorimeter EDS provides complete resolution of the TiL1 and TiLα peaks from O K, and separation of several of the numerous Ba M-family x-ray peaks. The difficult measurement situation imposed by the low fluorescence yield of the Ti L-family and the Ba M-family is obvious from the relative peak heights observed in Fig. 5(b). While mathematical deconvolution of the Si-EDS spectrum would be possible, the direct resolution of the peaks is much more desirable to achieve a robust analysis.

The improvement in the limit of detection that can be obtained with the microcalorimeter-EDS for the detection of Ba-like elements in BaTiO_3_ based on the spectrum shown in Fig. 5(b) is given in Table 4 [11]. The great improvement in the detection of the various peaks, including the Ba Mζ, in the microcalorimeter EDS spectrum compared to the Si-EDS spectrum renders the qualitative analysis far more reliable. While there is an improvement of only about a factor of 5 in *C*_DL_ due to the greater *P/B* of the microcalorimeter EDS, the limiting maximum spectral counting rate (about 800 c/s, full spectrum) of the microcalorimeter EDS reduces the improvement factor. The development of arrays of microcalorimeter EDS detector chips, each of which is capable of ≈ 1 kHz counting rate, will be a great aid to achieving useful low levels of detection for poorly excited elements [12] (Nam et al. 2001).

## 5. Summary

Low voltage microanalysis offers a great improvement in spatial resolution, both lateral and in depth, but at the expense of analytical flexibility. At an incident beam energy of *E*_0_ = 5 keV, useful characteristic peaks for analysis can be found for all elements in the Periodic Table, excepting H, He, and Li, providing a low over-voltage, *U* > 1.1 is acceptable. At lower beam energies, e.g., *E*_0_ = 2.5 keV, significant portions of the Periodic Table are not accessible even with *U* as low as 1.1. The choice of elemental peaks is further complicated by the low fluorescence yields of low photon energy L-shell and M-shell peaks. The relatively poor energy resolution of the semiconductor EDS leads to further limitations upon the spectral measurements, especially in situations of peak interference or of low concentration levels, where the low *P/B* of Si-EDS results in poor detection. Advances in x-ray spectrometry, including optics-augmented wavelength dispersive spectrometry and microcalorimeter energy dispersive spectrometry, hold considerable promise for improving the measurement situation to extend the practical applicability of low voltage microanalysis.

## Figures and Tables

**Table 1:** Range of x-ray excitation for CuK (K-edge, *E*_c_ = 8.98 keV) in various matrices in the conventional voltage regime

Matrix	25 keV	20 keV	15 keV	10 keV
C	6.3 μm	3.9 μm	1.9 μm	270 nm
Si	5.7 μm	3.5 μm	1.7 μm	250 nm
Fe	1.9 μm	1.2 μm	570 nm	83 nm
Au	1.0 μm	630 nm	310 nm	44 nm

**Table 2:** Range of x-ray excitation for Cu L (L-edge, *E*_c_=0.933 keV) in various matrices in the low voltagve regime

Matrix	5 keV	2.5 keV
C	490 nm	130 nm
Si	440 nm	120 nm
Fe	50 nm	40 nm
Au	80 nm	21 nm

**Table 3:** Predicted limits of detection for a Ba-like element in BaTiO_3_ with Si-EDS (*E*_0_ = 3 keV and 20 % deadtime)

	10 s	100s	1000 s	10 000 s
*C*_DL_	0.98	0.31	0.098	0.031

**Table 4:** Predicted limits of detection for a Ba-like element in BaTiO_3_ with the microcalorimeter-EDS (*E*_0_ = 3 keV and 20 % deadtime)

	10 s	100 s	1000 s	10,000 s
*C*_DL_	0.179	0.057	0.0179	0.0057
